# Outbreak of High-Risk Clone ST323 *Klebsiella pneumoniae* Resistant to Ceftazidime–Avibactam Due to Acquisition of *bla*_VEB-25_ and to Cefiderocol Due to Mutated *fiu* Gene

**DOI:** 10.3390/antibiotics14030223

**Published:** 2025-02-21

**Authors:** Irene Galani, Ilias Karaiskos, Maria Souli, Vassiliki Papoutsaki, Aikaterini Gkoufa, Anastasia Antoniadou, Helen Giamarellou

**Affiliations:** 1 Infectious Diseases Laboratory, 4th Department of Internal Medicine, National and Kapodistrian University of Athens, 12462 Athens, Greece; ananto@med.uoa.gr; 2 1st Internal Medicine & Infectious Diseases Department, Hygeia General Hospital, 15123 Athens, Greece; ikaraiskos@hygeia.gr (I.K.); katergouf@yahoo.gr (A.G.); e.giamarellou@hygeia.gr (H.G.); 3 Duke Clinical Research Institute, Duke University Medical Center, Durham, NC 27710, USA; msouli.md@gmail.com; 4 Infectious Diseases Laboratory, Hygeia General Hospital, 15123 Athens, Greece; vassiliki.papoutsaki@gmail.com; 5 Department of Infectious Diseases, First Department of Internal Medicine, National and Kapodistrian University of Athens, Laikon General Hospital, 11527 Athens, Greece; 6 Medical School, University of Cyprus, 2029 Nicosia, Cyprus

**Keywords:** *K. pneumoniae*, VEB-25, KPC-2, ceftazidime–avibactam resistant, cefiderocol resistant

## Abstract

**Background/Objectives:** The incidence of Ceftazidime/Avibactam (CZA)-resistant *Klebsiella pneumoniae* isolate co-producing *Klebsiella pneumoniae* carbapenemase 2 (KPC-2) and Vietnamese extended-spectrum β-lactamase 25 (VEB-25) has been on the rise in Greece over the past five years. This study investigates the isolation of ST323 *K. pneumoniae* isolates co-resistant to CZA and cefiderocol (FDC) from colonized and infected patients in a single hospital in Athens. **Methods:** CZA-resistant *K. pneumoniae* strains were isolated from 5 ICU patients from 27 December 2023 to 22 January 2024. Antimicrobial susceptibility was tested against a panel of agents. Whole-genome sequencing of the isolates was carried out to identify the acquired resistance genes and mutations that were associated with CZA and FDC resistance. **Results:** The *K. pneumoniae* isolates belonged to ST323 and harbored *bla*_KPC-2_ and *bla*_VEB-25_. The isolates had a minimum inhibitory concentration (MIC) of >256 mg/L for CZA and 32 mg/L for FDC, due to the disrupted catecholate siderophore receptor Fiu. *bla*_VEB-25_ was located on an IncC non-conjugative plasmid and on a ~14 kb multidrug resistance (MDR) region comprising 15 further acquired resistance genes. Transformation studies showed that the *bla*_VEB-25_-carrying plasmid provided resistance to most of the β-lactams tested, including CZA. The isolates remained susceptible to carbapenems, imipenem/relebactam, and meropenem/vaborbactam. The plasmid harbored the citrate-dependent iron (III) uptake system (*fecIRABCDE*), which increased the MIC of FDC from ≤0.08 mg/L to 2 mg/L. **Conclusions:** The *bla*_VEB-25_ gene was associated with IncC plasmids which are important contributors to the spread of key antibiotic resistance genes. Strict infection control measures must be elaborated upon to prevent the spread of extensively drug-resistant organisms such as those described here.

## 1. Introduction

Antibiotic resistance is a dynamic and evolving process among bacteria, primarily driven by prolonged exposure to antimicrobial agents. This underscores the necessity for continuous surveillance to enable early detection of resistance mechanisms, leveraging advanced genetic assays and molecular technologies [1]. Such efforts are particularly critical following the commercial approval of novel β-lactam–β-lactamase inhibitor (BLBLI) combinations. These agents have demonstrated high clinical efficacy, and a more favorable adverse event profile compared to older antibiotics, thereby enhancing therapeutic outcomes of infections caused by carbapenem-resistant pathogens [2].

*Klebsiella pneumoniae* is a frequent cause of healthcare-associated infections including pneumonia, urinary tract, wound, and bloodstream infections [3]. Hospital infections caused by carbapenemase-producing *K. pneumoniae* (CPKP) are a worldwide problem with high treatment failure and mortality rates.

The worldwide spread of CPKP is driven by the transmission of international high-risk clones in healthcare facilities [4,5]. In Greece, Tryfinopoulou et al. reported the dissemination of high-risk clones of CPKP and showed the persistent spread of previously established (ST258, ST11, and ST147) as well as newly emerging high-risk clones (ST39 and ST323) in Greek hospitals over a 10-year period [6]. ST323 has been mainly associated with the *bla*_CTX-M-15_ extended-spectrum β-lactamase gene and has been linked to healthcare-associated infections in Australia and Poland [7,8] or community-acquired urinary tract infections in Tunisia [9]. Sporadic cases of ST323 CPKP carrying *bla*_VIM_ or both *bla*_KPC-2_ and *bla*_VIM_ have been reported in Greece in 2005 and 2011 [10,11], and Zarras et al. reported the isolation of such isolates from three ICU patients in Thessaloniki in 2019 [12]. ST323 was not detected in the carbapenem- and/or colistin-resistant Enterobacterales (CCRE) survey conducted in 2019 in 15 Greek hospitals. However, this high-risk clone had rapidly spread across six Greek hospitals in Attica, Peloponnese, and Crete by 2022. Interestingly, this clone was not found in the six participating hospitals in northern and central Greece [6].

VEB-25 (Vietnamese extended-spectrum β-lactamase) was first reported in 2019 in two KPC-KP isolates from patients in two Greek hospitals [13] and caused an outbreak in two ICUs of a hospital in Athens. This outbreak affected seven patients over five weeks and was linked to ceftazidime/avibactam (CZA) resistance [14]. The isolates belonged to ST147 and ST258. Later, Zarras et al. reported a case of neonatal sepsis due to CZA-resistant KPC-KP carrying *bla*_VEB-25_ belonging to ST35 that was successfully treated with non-conventional “off-label” antimicrobial agents [15]. Furthermore, in 2023, Findlay et al. reported the isolation of an ST323 KPC-producing *Klebsiella pneumoniae* (KPC-KP) resistant to CZA due to the acquisition of VEB-25. The source was a patient in Switzerland who had previously been hospitalized in Greece [16]. VEB-type β-lactamases are a group of Ambler class A enzymes inhibited by avibactam, although VEB-25 differs from VEB-1 by a substitution of lysine by an arginine at position 237 (K234R, per Ambler numbering scheme), due to a nt A710G substitution [13], which compromises the inhibitory efficiency of avibactam [17]. To our knowledge, this variant has only been reported in Greece and in one patient in Switzerland who was epidemiologically linked with Greece.

Cefiderocol (FDC) is a new siderophore cephalosporin that penetrates the outer membrane of Gram-negative bacteria using their iron transport system, binds to penicillin-binding proteins (PBPs), and inhibits the bacterial wall synthesis [18]. The activity of FDC against aerobic Gram-negative bacilli is equal to or superior to that of CZA and meropenem due to its high stability against β-lactamases including ESBLs, AmpC, and carbapenemases. In a recent Greek study, FDC inhibited 80% of the CPKP isolates with minimum inhibitory concentrations (MICs) ranging from 0.006 to 32 mg/L, and the concentration of FDC inhibiting 90% (MIC_90_) of the isolates was 4 mg/L [19]. FDC was approved by the US Food and Drug Administration and the European Medicines Agency in 2020 to treat Enterobacterales, *Acinetobacter baumannii,* and *Pseudomonas aeruginosa* invasive infections caused by carbapenem- and CZA-resistant strains [20,21]. However, despite the initial promising in vitro and in vivo results, the emergence of resistance has been observed worldwide. The mechanisms underlying FDC resistance remain poorly described. Combined factors such as modification of the target (PBP-3), mutations in iron transporters (mainly *cirA* and *fiu*), expression of β-lactamases, porin loss, or efflux pump overexpression by some isolates have been reported [22]. Additionally, the acquisition of a transferable extrachromosomal *fec* operon has been associated with a cefiderocol MIC increase in Enterobacterales [23,24]. Unfortunately, this *fec* gene cluster is present in many *K. pneumoniae* strains, including those isolated before the introduction of FDC in clinical therapy [24]. According to Polani et al., in the future, FDC may act as a positive selector for plasmids carrying the *fec* genes, for which prevalence can be expected to increase [24].

We report the isolation of ST323 carbapenem-resistant *K. pneumoniae* isolates co-resistant to CZA, due to VEB-25 production, and FDC from colonized and/or infected patients in a single hospital in Athens. FDC was introduced in Greece in 2024 and is supplied by the Institute of Pharmaceutical Research & Technology (IFET), which imports and distributes essential pharmaceutical products to the Greek market. Since no pharmaceutical company has officially launched FDC in the country, IFET ensures its availability for clinical use. The increasing use of CZA and FDC is expected to further elevate the clinical significance of VEB-25- and *fec* gene cluster-mediated resistance. Notably, these resistance determinants are located on the same plasmid, which has already been acquired by the high-risk CPKP clone. This raises significant concerns about the potential emergence of pan-drug resistance, posing a serious threat to antimicrobial treatment options.

## 2. Results

### 2.1. Antibiotic Susceptibility Testing and Molecular Investigation of the Isolates

Susceptibility testing evaluated according to the European Committee on Antimicrobial Susceptibility Testing (EUCAST) clinical breakpoints [25] revealed that *K. pneumoniae* isolates were resistant to all β-lactams tested, including carbapenems, FDC, and the BLBLI combinations CZA and aztreonam/avibactam (AZA). The isolates remained susceptible to imipenem/relebactam (IMR) (MIC 0.5–1 mg/L) and meropenem/vaborbactam (MEV) (MIC 0.125–0.25 mg/L) (Table 1). All harbored *bla*_KPC-2_ as the sole carbapenemase gene, as initially detected with NG Test CARBA 5 and confirmed by PCR and sequencing (Table 1). Additionally, all isolates harbored the *bla*_VEB-25_ variant, which explained the CZA resistance.

All isolates exhibited an identical pulsed-field gel electrophoresis pulsotype using enzyme *Spe*I and conditions as previously described [26]. Representative isolates were subjected to whole-genome sequencing (WGS), which identified that the isolates belonged to clonal lineage ST323 and harbored *bla*_KPC-2_, *bla*_VEB-25_, and 15 further acquired resistance genes encoding resistance to multiple antibiotic classes: *tet(G)* (tetracycline efflux pump), *qnrS1* (quinolones), *cmlA5* (phenicols), *arr-2* (rifiampicin), *dfrA23* (trimethoprim), *sul1* and *sul2* (sulphonamides), and seven aminoglycoside resistance genes (*rmtB1*, *aph(3″*)-*Ia*, *ant*(*2″*)-*Ia*, *ant*(*3″*)-*Ia*, *aph*(*6*)-*Id* and *aph*(*3″*)-*Ib*) (Table 2).

The isolates had an intact OmpK35 and an OmpK36_v6 variant [27]. OmpK36_v6 exhibited a substitution of amino acids, Ala267-Gly268-Ser269-Leu270 to Phe267-Ser268-Gly269-Asn270, and an insertion of amino acids, Glu272-Ser273-Asp274-Ser275-Ile276-Ser277-Gly278, in loop L6, an external loop, facing the cell exterior.

A frameshift mutation in the *fiu* gene (insertion of an adenine after 652 nucleotides) and a subsequent disruption of the coding catecholate siderophore receptor Fiu protein, due to a stop codon after amino acid 250, were also found, contributing to FDC resistance [22,28].

No mutations were found in *ramA* or *acrR* genes (tigecycline resistance determinant genes); however, the amino acid replacement of N87D in GyrA occurred in both isolates, contributing to quinolone resistance.

Plasmid replicon type analyses revealed a number of replicon types present, comprising Col440II, ColRNAI, IncC, IncFIB(pQIL), IncFII(K), IncFII(pKP91), IncR, and repB(R1701) (Table 2).

Conjugation experiments were unsuccessful; however, *E. coli* TOP10 transformants harboring the *bla*_VEB-25_-carrying plasmid were confirmed by susceptibility testing and subsequent amplification and sequencing of the *bla*_VEB-25_ gene.

*E. coli* TOP10 transformants harboring *bla*_VEB-25_ were resistant to most of the β-lactams tested, including the BLBLI combinations CZA and AZA. The isolates remained susceptible to FDC, carbapenems, IMR, and MEV (Table 1). The *bla*_VEB-25_ gene was located on an IncC plasmid in a ~14 kb multidrug resistance region comprising IS*10A*, *bla*_VEB-25_, *aadB*, *arr-2*, *cmlA5*, *bla*_OXA-10_, *aadA1*, *qacE*Δ*1*, Δ*sul1*, *floR2*, *tetR*, *tet*(G), *lysR*, IS*CR3*-like, *groEL*, IS*L3*, *rmtB1,* and *bla*_TEM-1_. This region was similar to the ~14 kb MDR region previously described by Voulgari et al., while the plasmid was similar to N3418 plasmid pVEB-25_IncC (GenBank: OQ362291.1) described by Findlay et al. [13,16], with most of the differences found in this MDR region, due to the lack of a ~5 kb region that includes *rmtB1* and *bla*_TEM-1_. Similar to N3418 plasmid pVEB-25_IncC, the plasmid in this study harbored the mercury resistance operon (*merACPTR*), the HigAB type II toxin–antitoxin system (typical of IncC plasmids), and the citrate-dependent iron (III) uptake system (*fecIRABCDE*), which has recently been linked to reduced susceptibility to FDC [23]. FDC MICs increased from the initial ≤ 0.08 mg/L (MIC of *E. coli* TOP10) to 2 mg/L in both transformants (Table 1).

### 2.2. Epidemiologic Investigation of the Outbreak

In four of the patients in this study, colonization by the CZA-resistant KPC-KP isolate followed prior exposure to carbapenem therapy. Three of the five colonized patients presented with a septic episode caused by the same isolate (Patients 1, 3, and 4 in Table 3) and the fourth patient by a KPC-KP isolate susceptible to CZA and to all aminoglycosides (Patient 2 in Table 3).

Novel BLBLIs were utilized for the treatment of the infectious episodes in these patients. Specifically, three patients received meropenem–vaborbactam, while one patient was treated with imipenem–cilastatin–relebactam. These therapies were administered for conditions including ventilator-associated pneumonia (VAP) and bloodstream infection (BSI) associated with central venous catheter (CVC) use. Source control strategies played a pivotal role in infection management for two patients. All patients were treated promptly with novel BLBLIs; however, 28-day mortality was 50% in the infected patients. Given the small sample size, these findings should be interpreted with caution. The observed 50% mortality rate is likely influenced by severe underlying conditions, prolonged hospitalization, and critical illness, aligning with previous reports of up to 60% mortality for CZA–AVI-resistant infections [29]. The baseline characteristics of these patients are outlined in Table 3.

A thorough investigation was conducted to trace the source and transmission pathways of the outbreak. Epidemiologic data were gathered, including patient histories, antimicrobial usage, and infection timelines. The spatial layout and patient flow within the ICUs were reviewed to identify potential points of environmental contamination or breaches in infection control practices. The outbreak was successfully contained by a combination of intensified infection control measures and rigorous adherence to infection prevention protocols. These included isolating colonized patients in single rooms, enhancing implementation of personal protective equipment protocols, and dedicating resources to environmental cleaning and surveillance of hand hygiene compliance. The absence of new cases after January 2024 indicates the effectiveness of these interventions.

## 3. Discussion

CZA demonstrates high in vitro activity against non-metallo-β-lactamase-producing *K. pneumoniae* strains [26], although VEB-mediated resistance has been well documented in Greece [13,14,15]. Hopefully, only rare cases have been reported so far, as rare cases of CZA resistance among KPC-producing isolates, endowed with other resistance mechanisms, have also been reported.

In most cases reported so far, and in the cases reported in this study, *bla*_VEB-25_ is located in a ~14 kb multidrug resistance (MDR) region comprising IS*10A*, *bla*_VEB-25_, *aadB*, *arr-2*, *cmlA5*, *bla*_OXA-10_, *aadA1*, *qacE*Δ*1*, Δ*sul1*, *floR2*, *tetR*, *tet*(G), *lysR*, IS*CR3*-like, *groEL*, IS*L3*, *rmtB1,* and *bla*_TEM-1_ [13]. In only one case reported in Switzerland, from a patient who had previously been hospitalized in Greece, this MDR region was smaller (~9 kb), lacking ~5 kb that include *rmtB1* and *bla*TEM-1 [16]. The MDR region is always located on IncC plasmids, conjugative [13,14] or not [16], in different sequence types of *K. pneumoniae* isolates (ST147, ST258, ST35, and ST323) that always produce KPC (mostly KPC-2).

Previous epidemiologic analyses indicated that a conjugative plasmid co-harboring *bla*_VEB-1_, *bla*_OXA-10_, *bla*_TEM-1B,_ and *rmtB* is present in 8% of all *bla* _KPC_-positive isolates in Greece [30], while among aminoglycoside-resistant *K. pneumoniae* isolates, 37.8% harbored a 16S rRNA methylase-coding gene, mainly *rmtB*, located on an Inc A/C plasmid along with *bla*_VEB_, *bla*_OXA-10_, and *bla*_TEM_ [31].

The backbone of the *IncC* plasmids in this study, carrying the MDR region, was similar to that of N3418 plasmid pVEB-25_IncC (GenBank: OQ362291.1) described by Findlay et al. [16], harboring the mercury resistance operon (*merACPTR*), typical of IncC plasmids’ HigAB type II toxin–antitoxin system and the citrate-dependent iron (III) uptake system (*fecIRABCDE*).

Additionally, the *K. pneumoniae* isolates in this study, similar to the N3418 isolate reported in Switzerland [16], belong to ST323, a sequence type that was rapidly spread in Greek hospitals in 2022 [6].

The MDR region carrying the *bla*_VEB-25_ gene is an already-established vehicle that enhances dissemination of VEB-mediated CZA resistance in KPC-KP of different sequence types in Greek hospitals. The emergence of CZA resistance dramatically limits treatment options against carbapenemase-producing Enterobacterales. In this report, infected patients were treated with novel BLBLIs; however, 28-day mortality was 50% among infected patients.

Finally, ST323 isolates in this study and the N3418 isolate from Switzerland were resistant to the recently approved cefiderocol with MICs ranging from 32 to 64 mg/L due to *fiu* chromosomal gene disruption and to the presence of *fecIRABCDE* gene cluster in the IncC plasmid. The deletion of *cirA* or *fiu* in an *E. coli* isolate increased the MIC of FDC within 2-fold of that required for the parent strain, while double knockout of *cirA* and *fiu* was needed to increase the MIC of cefiderocol 16-fold [28]. In the isolates presented in this study, *cirA* was intact and only *fiu* had a frameshift mutation resulting in a smaller protein. Furthermore, the isolates had an intact OmpK35 and an OmpK36_v6 variant which was previously associated with the unrelated ST323 and ST383 *K. pneumoniae* isolates from Greece, affecting the entry of β-lactams [27,32]. This variant of OmpK36 must be further investigated for a possible contribution to FDC resistance. Additionally, the presence of the plasmidic *fecIRABCDE* gene cluster, which increased the MIC of FDC in the *E. coli* transformants by at least 5-fold (from ≤0.08 mg/L to 2 mg/L), may contribute synergistically to the increased FDC MICs (32–64 mg/L) observed in the *K. pneumoniae* isolates.

The acquisition of the transferable extrachromosomal *fec* operon has been associated with a cefiderocol MIC increase in Enterobacterales [23,24], and recently, Polani et al. reported that the *fec* gene cluster is present in many *K. pneumoniae* strains, including those isolated before the introduction of FDC in clinical therapy [24]. Although Poirel et al. have reported that VEB-1 does not hydrolyze FDC [33], the hydrolytic activity of VEB-25 against FDC must be investigated. Previous studies that reported VEB-25-producing *K. pneumoniae* isolates have not determined the MIC for FDC [13,14,15].

Here, we report the co-emergence of resistance to CZA due to VEB-25 production and to FDC due to mutated *fiu* and co-existence of the *fec* gene cluster in the successful high-risk clone ST323. This is a public health problem that needs to be monitored and also poses a challenge for clinical microbiology laboratories. Neither of the patients was previously treated with CZA or FDC, indicating that this clone is likely circulating in Greece.

In conclusion, the dissemination of VEB-25 among multidrug-resistant organisms is an unwelcome event. The *bla*_VEB-25_ gene was associated with IncC plasmids which are important contributors to the spread of key antibiotic resistance genes. The co-presence of the *fec* cluster in the same plasmid is even more worrisome. As already reported, the use of FDC may also act as a positive selector for plasmids carrying the *fec* genes, for which prevalence can be expected to increase [24]. Our findings highlight the importance of genomic data surveillance to detect onward pathogen and plasmid transmission and persistence. Strict infection control measures must be elaborated upon to prevent the spread of extensively drug-resistant organisms such as those described here that co-produced KPC-2 and VEB-25 and were also resistant to cefiderocol.

## 4. Materials and Methods

### 4.1. Detection of the Outbreak

This study was conducted at a hospital comprising two mixed ICUs located in separate areas. ICU-1 includes a 14-bed open space and four single-patient rooms, while ICU-2 consists of an 11-bed open space and five single-patient rooms. In this case, routine rectal surveillance cultures were instrumental in identifying carbapenemase-producing bacteria among patients in the ICUs. These cultures were performed biweekly on all ICU patients. The outbreak was identified when five patients tested positive for colonization with KPC-KP which were resistant to CZA.

### 4.2. Bacterial Isolates

Eight KPC-KP isolates, resistant to CZA, were sent to the Infectious Diseases Laboratory of the 4th Dept of Internal Medicine, Faculty of Medicine, National and Kapodistrian University of Athens, for further testing. The strains were isolated from five ICU patients during the period between 27 December 2023 and 22 January 2024. All five patients were colonized by a CZA-resistant KPC-KP strain and three of them developed an infection from this isolate.

### 4.3. Susceptibility Testing

Species identification and primary determination of MICs were performed by Vitek^®^2 (bioMérieux, Marcy-l’Etoile, France). Carbapenemase production was defined by the multiplex immunochromatographic assay NG Test CARBA 5 (NG Biotech, Guipry, France). Susceptibility testing of FDC and AZA was evaluated by disk diffusion, while MICs of FDC and colistin were determined by the commercially available broth microdilution method ComASP according to the manufacturer’s instructions. Ceftazidime, CZA, meropenem, MEV, IMR, amikacin, gentamicin, tigecycline, fosfomycin, and chloramphenicol MICs were also determined by Liofilchem^®^ MIC Test Strips (Liofilchem srl, Roseto degli Abruzzi, Italy) according to the manufacturer’s instructions. *Escherichia coli* ATCC 25922, *E. coli* NCTC 13846, and *K. pneumoniae* ATCC 700603 were used as quality control strains. Antimicrobial susceptibility results were interpreted according to the EUCAST recommendations [25].

### 4.4. DNA Extraction, Genome Sequencing, and Data Analysis

DNA was extracted from *K. pneumoniae* isolates using the PureLink^®^ Genomic DNA mini kit (Life Technologies, Invitrogen^TM^, Carlsbad, CA, USA) following the manufacturer’s Gram-Negative Bacterial Cell DNA extraction protocol. Libraries were prepared using Ion Torrent technology and Ion Chef workflows (Thermo Fisher Scientific, Waltham, MA, USA). Sequencing was performed in the S5XLS system and analysis of primary data was conducted with Ion Torrent Suite v.5.10.0. The raw reads were assembled using the SPAdes assembler available from the Bacterial and Viral Bioinformatics Resource Center (BV-BRC), accessed on 12 April 2024, using default settings [34]. Automatic annotation of the bacterial genome was performed with RASTtk available from the BV-BRC [34]. Assembled contigs were also submitted to the Center for Genomic Epidemiology platform for species identification (KmerFinder 3.2), Multilocus Sequence Typing (MLST 2.0), detection of antimicrobial resistance genes (ResFinder 4.6.0), identification of plasmid incompatibility groups (PlasmidFinder 2.1), and plasmid MLST (pMLST 2.0), accessed on 13 September 2024 [35]. Capsule and lipopolysaccharide serotype were predicted by the Kaptive Web tool, accessed on 13 September 2024 [36]. Selected genes were also aligned to reference genes with the NCBI BLAST algorithm version BLAST+ 2.16.0 [37].

### 4.5. Transferability of bla_VEB-25_ Genes

Conjugation of the *bla*_VEB-25_-harboring plasmids was attempted using a rifampicin-resistant *E. coli* RC85 R K12 as a recipient strain, as previously described [14], with selection on rifampicin at 512 mg/L and ceftazidimeat at 256 mg/L or gentamicin at 50 mg/L, with no success.

Transformation of competent *E. coli* TOP10 cells (Invitrogen, Milan, Italy) was performed by plasmid DNA that was extracted (PureLink HiPure plasmid filter midiprep kit; Invitrogen, Milan, Italy) from all eight VEB-25-producing isolates. Transformants were selected on Luria Bertani agar (Sigma-Aldrich Inc., St. Louis, MO, USA) plates containing 256 mg/L ceftazidime. Transformants were subjected to antibiotic susceptibility testing and PCR with specific primers to determine the presence of *bla* genes.

## Figures and Tables

**Table 1:** Antimicrobial susceptibility and genotypic characteristics of the *Klebsiella pneumoniae* isolates and their transformants.

Patient (No., Sex, Age)	1		2	3		4		5	Laboratory Strain	
	Male		Female	Male		Male		Male		
	75		71	46		79		77		
Isolate	1-R.2	1-S.7	2-R.1	3-R.5	3-BL.6	4-R.8	4-BS.3	5-R.4	TOP10/pl-VEB-25 *	TOP10
Source	Rectal	Sputum	Rectal	Rectal	Blood	Rectal	Bronchial secretions	Rectal	Transformant	Laboratory strain
Day of isolation	27 December 2023	9 January 2024	2 January 2024	2 January 2024	22 January 2024	22 January 2024	10 January 2024	22 January 2024	-	-
MLST-type	ST323	ST323	ST323	ST323	ST323	ST323	ST323	ST323	ND	ND
KPC-type	KPC-2	KPC-2	KPC-2	KPC-2	KPC-2	KPC-2	KPC-2	KPC-2	None	None
VEB-type	VEB-25	VEB-25	VEB-25	VEB-25	VEB-25	VEB-25	VEB-25	VEB-25	VEB-25	None
Antibiotics tested	in mg/L									
Penicillins										
Amoxicillin/ Clavulanate	>32	>32	>32	>32	>32	>32	>32	>32	>32	≤4
Ampicillin/ Sulbactam	>16	>16	>16	>16	>16	>16	>16	>16	>16	4
Piperacillin/ Tazobactam	>64	>64	>64	>64	>64	>64	>64	>64	64	≤4
Cephalosporins										
Cefuroxime	>32	>32	>32	>32	>32	>32	>32	>32	>32	4
Cefoxitin	>32	>32	>32	>32	>32	>32	>32	>32	8	8
Cefotaxime	>32	>32	>32	>32	>32	>32	>32	>32	32	≤0.25
Ceftazidime	>256	>256	>256	>256	>256	>256	>256	>256	>256	0.25
Ceft/avi	>256	>256	>256	>256	>256	>256	>256	>256	>256	0.25
Ceftriaxone	>32	>32	>32	>32	>32	>32	>32	>32	>32	≤0.25
Ceft/taz	>16	>16	>16	>16	>16	>16	>16	>16	>16	≤0.25
Cefepime	>16	>16	>16	>16	>16	>16	>16	>16	8	≤0.12
Cefiderocol	32	32	32	32	32	32	32	32	2	0.016
Monobactams										
Aztreonam	>32	>32	>32	>32	>32	>32	>32	>32	>32	≤1
Aztreonam/Avibactam **	R	R	R	R	R	R	R	R	R	S
Carbapenems										
Ertapenem	>4	>4	>4	>4	>4	>4	>4	>4	≤0.12	≤0.12
Imipenem	>8	>8	>8	>8	>8	>8	>8	>8	≤0.25	≤0.25
Imipenem/ Relebactam	0.5	0.5	0.5	0.5	1	0.5	1	0.5	0.5	0.25
Meropenem	>8	>8	>8	16	16	>32	>32	>8	0.032	0.032
Meropenem/ Vaborbactam	0.25	0.25	0.25	0.125	0.125	0.25	0.25	0.25	0.032	0.032
Aminoglycosides										
Amikacin	>256	>256	>256	>256	>256	>256	>256	>256	>256	2
Gentamicin	>256	>256	>256	>256	>256	>256	>256	>256	>256	0.5
Tobramycin	>8	>8	>8	>8	>8	>8	>8	>8	>8	≤1
Tetracyclines										
Tigecycline	>4	>4	>4	>4	>4	>4	>4	>4	0.25	0.25
Miscellaneous agents										
Fosfomycin	64	64	64	64	64	64	64	64	16	0.25
Colistin	0.5	0.5	0.5	0.5	0.5	>4/2	>4/2	0.5	≤0.25	≤0.25
Trimethoprim/ Sulphamethoxazole	>160	>160	>160	>160	>160	>160	>160	>160	>160	≤20
Chloramphenicol	128	128	64	128	128	64	64	64	16	2

ND, not defined; R, resistant; S, susceptible. * All transformants had identical susceptibilities. ** Susceptibility to Aztreonam/Avibactam was determined by disk diffusion method. All resistant isolates exhibited disk diameters of 17–18 mm, while the susceptible laboratory strain TOP10 exhibited a zone diameter of 34 mm.

**Table 2:** Acquired antibiotic resistance genes and plasmid replicons of *Klebsiella pneumoniae* clinical isolates and their transformants.

Isolate	4-BS.3	TOP10/pl3	3-BL.6	TOP10/pl6
Source	Bronchial secretions	Transformant	Blood	Transformant
Day of isolation	10 January 2024	-	22 January 2024	-
Acquired resistance genes				
β-lactamase genes	*bla*_SHV-11_, *bla*_KPC-2_, *bla*_VEB-25_, *bla*_OXA-10_, *bla*_TEM-1B_	*bla*_KPC-2_, *bla*_VEB-25_, *bla*_OXA-10_, *bla*_TEM-1B_	*bla*_SHV-11_, *bla*_KPC-2_, *bla*_VEB-25_, *bla*_OXA-10_, *bla*_TEM-1B_	*bla*_KPC-2_, *bla*_VEB-25_, *bla*_OXA-10_, *bla*_TEM-1B_
Aminoglycoside-modifying enzyme-coding genes	*aph(6)-Id*, *aph(3″)-Ib*, *aadA1*, *ant(2″)-Ia*, *rmtB*	*aph(6)-Id*, *aph(3″)-Ib*, *aadA1*, *ant(2″)-Ia*, *rmtB*	*aph(6)-Id*, *aph(3″)-Ib*, *aadA1*, *ant(2″)-Ia*, *aph(3′)-Ia*, *rmtB*	*aph(6)-Id*, *aph(3″)-Ib*, *aadA1*, *ant(2″)-Ia*, *aph(3′)-Ia*, *rmtB*
Quinolones	*qnrS1*	*qnrS1*	*qnrS1*	*qnrS1*
Tetracycline	*tet(A)*, *tet(G)*	*tet(A)*, *tet(G)*	*tet(A)*, *tet(G)*	*tet(A)*, *tet(G)*
Fosfomycin	*fosA7*, *fosA6*		*fosA7*, *fosA6*	
Trimethoprim	*dfrA23*	*dfrA23*	*dfrA23*	*dfrA23*
Phenicol	*cmlA5*	*cmlA5*	*cmlA5*	*cmlA5*
Sulfonamide	*sul1*, *sul2*	*sul1*, *sul2*	*sul1*, *sul2*	*sul1*, *sul2*
Plasmid replicons				
Plasmid replicons	Col440II, ColRNAI, IncC, IncFIB(pQil), IncFII(K), IncFII(pKP91), IncR, repB(R1701)	IncC	Col440II, ColRNAI, IncC, IncFIB(pQil), IncFII(K), IncFII(pKP91), IncR, repB(R1701)	IncC

**Table 3:** Baseline characteristics of the patients colonized by a CZA-R KPC-*K. pneumoniae* isolate.

A/A Patient	Gender, Age	Charlson Comorbidity Index	Cause of ICU Admission	Date of Colonization by CZA-R KPC-KP Isolate	Antibiotics Prescribed Prior to Colonization	Prior Carbapenem Use (Days)	Type/Date of Infection for BLBLI Administration	Antimicrobial Treatment	Previous Treatment with BLBLI	Treatment Outcome	28-Day Outcome
1	Male, 75	9	Respiratory failure	27 December 2024	CRO, TZP, MFX	No	VAP by CZA-R KPC-KP/9 January 2024	MER/VAB	No	Reduction of ventilation support, transfer to rehabilitation center	Death on 14th day after infection
2	Female, 71	7	Brain injury/Subdural hematoma	2 January 2024	VAN, TZP, CRO	Yes (7)	CRBSI by KPC-KP/9 January 2024	MER/VAB	No	Sterile blood cultures, removal of central line	Alive
3	Male, 46	1	Ischemic stroke	2 January 2024	CRO, TZP, VAN, MEM	Yes (3)	CRBSI by CZA-R KPC-KP/22 January 2024	IMI/REL	No	Sterile blood cultures, removal of central line	Alive
4	Male, 79	8	Respiratory failure	8 January 2024	TZP, VAN, MEM	Yes (13)	VAP by CZA-R KPC-KP/10 January 2024	MER/VAB	No	Complicated with super infection with CRAB	Death on 10th day after infection
5	Male, 77	9	Chest injury	22 January 2024	LZD, PZT, MEM	Yes (3)	-	-	No	-	Death occurred within 24 h after BSI with KPC-*K. pneumoniae* susceptible to CZA

Abbreviations: BLBLI: β-lactam/β-lactamase inhibitor; BSI: bloodstream infection; CRBSI: catheter-related bloodstream infection; CRO: ceftriaxone; IMI/REL: imipenem–cilastatin–relebactam; ICU: intensive care unit; LZD: linezolid; MEM: meropenem; MER/VAB: meropenem–vaborbactam; MFX: moxifloxacin; TZP: piperacillin–tazobactam; VAN: vancomycin; VAP: ventilator-associated pneumonia.

## Data Availability

The whole genome sequencing data for isolates 4-BS.3 and 3-BL.6 have been deposited in the NCBI under BioProject accession number PRJNA1215006 and BioSample accession numbers SAMN46391955 and SAMN46391956.
